# Nursing quality control combined with intensified psychological care reduces emotional distress and improves clinical outcomes in patients undergoing blood purification

**DOI:** 10.23938/ASSN.1109

**Published:** 2025-09-18

**Authors:** Xiao-Yun Cao, Ruo-Yu Wang, Jin-Feng Xue, Xing-Hong Miao

**Affiliations:** 1 Hospital of Jiangnan University Wuxi Jiangsu Province China; 2 Nantong Second People’s Hospital Satisfaction Service Center Nantong Jiangsu Province China

**Keywords:** Dialysis, Nursing, Quality of Health Care, Emotions, Randomized Control Trial, Diálisis, Enfermería, Calidad Asistencial, Emociones, Ensayo Clínico Aleatorizado

## Abstract

**Background:**

This study aimed to evaluate the effects of nursing quality control combined with intensified psychological care on negative emotions and health outcomes in patients undergoing blood purification.

**Methods:**

Patients who underwent blood purification at the Affiliated Hospital of Jiangnan University (China) from January 2021 to December 2023 were enrolled and assigned to either a control or an intervention group using a random number table. Both groups received standard care, while the intervention group additionally received nursing quality control and intensified psychological care. Outcomes compared between groups included negative emotions (assessed by the Hamilton Anxiety Scale and Hamilton Depression Scale scores), quality of life (SF-36), treatment adherence, and complication rates.

**Results:**

Prior to the treatment, both groups (150 patients in each) were comparable in the Hamilton Anxiety Scale, the Hamilton Depression Scale, and SF-36 scores. After the treatment, the intervention group showed significantly greater reduction in the Hamilton Anxiety Scale (15.89 vs. 7.19) and Hamilton Depression Scale (12.22 vs. 4.74) scores, and greater improvements in SF-36 scores, particularly in physical functioning (37.99 vs. 22.61) and mental health (34.48 vs. 18.95). Moreover, treatment adherence was significantly higher in the intervention group (98.67% vs 70%), while the complication rate was markedly lower (10% vs. 35.33%). All differences were statistically significant (p < 0.001).

**Conclusions:**

Nursing quality control combined with intensified psychological care significantly improves negative emotional states and quality of life in patients undergoing blood purification, while also enhancing treatment adherence and reducing the incidence of complications.

## RESUMEN

**Fundamento:**

Evaluar los efectos de combinar el control de calidad asistencial enfermera con atención psicológica intensificada sobre emociones negativas y resultados de salud en pacientes sometidos a purificación sanguínea.

**Métodos:**

Se incluyeron pacientes sometidos a purificación sanguínea en el *Affiliated Hospital of Jiangnan University* (China) entre enero de 2021 y diciembre de 2023. Mediante una tabla de números aleatorios, fueron asignados al grupo control o al grupo intervención. Ambos grupos recibieron la atención estándar y el grupo intervención también recibió control de calidad de enfermería combinado con atención psicológica intensificada. Se compararon las emociones negativas (ansiedad y depresión, evaluadas con HAMA y HAMD), la calidad de vida (SF-36), la adherencia al tratamiento y la tasa de complicaciones.

**Resultados:**

Antes de la intervención, ambos grupos de 150 pacientes eran comparables respecto a las puntuaciones de HAMA, HAMD y SF-36. Tras la intervención, el grupo intervención mostró mayores reducciones en las puntuaciones de HAMA (15,89 vs 7,19) y HAMD (12,22 vs 4,74), así como mejoras más marcadas en la calidad de vida, especialmente en Función Física (37,99 vs 22,61) y Salud Mental (34,48 vs 18,95). Además, presentó una mayor adherencia al tratamiento (98,67% vs 70%) y una menor tasa de complicaciones (10% vs 35,33%). Todas las diferencias fueron estadísticamente significativas (p<0,001).

**Conclusiones:**

El control de calidad en enfermería, combinado con atención psicológica intensificada, mejora las emociones negativas y la calidad de vida en pacientes sometidos a purificación sanguínea, favoreciendo la adherencia al tratamiento y reduciendo las complicaciones.

## INTRODUCTION

Blood purification is a critical medical intervention that uses physical, chemical, and biological methods to remove excess water and toxins from the bloodstream. It is widely employed in the treatment of conditions such as kidney diseases (e.g., uremia), poisoning, liver failure, and autoimmune disorders, serving as an essential therapeutic modality alongside pharmacological and surgical interventions^1^. The procedure involves extracting the patient’s blood, purifying it using specialized equipment, and reinfusing it into the body. This technique helps restore fluid-electrolyte and acid-base balance, regulates immune function, and effectively alleviates clinical symptoms^2^. Common methods include hemodialysis, hemofiltration, hemodiafiltration, hemoperfusion, plasma exchange, and immunoadsorption.

Despite its efficacy, blood purification is a complex and high-risk procedure, exposing patients to potential complications such as infections and dialysis disequilibrium. These risks necessitate rigorous, scientifically guided nursing care to ensure patient safety and minimize adverse outcomes^3^.

In clinical practice, standard nursing protocols are widely applied; however, they often fail to address the emotional challenges experienced by patients undergoing prolonged and intensive treatments. Many of these patients suffer from adverse emotional states, such as anxiety and depression, which negatively affect treatment adherence, quality of life, and overall health outcomes. This emotional distress not only hinders recovery but also increases the burden on healthcare systems and complicates the delivery of effective nursing care.

While existing literature highlights the importance of psychological interventions in reducing emotional distress and enhancing compliance, few studies have examined the integration of psychological care with systematic nursing quality control^4^^,^^5^. Nursing quality control ensures high standards of care and reduces procedural risks, while psychological care helps patients manage stress and improve their overall treatment experience. However, limited research has evaluated the combined effect of these two approaches on patients undergoing blood purification^6^^,^^7^.

This study aims to address this gap by evaluating the effectiveness of nursing quality control combined with intensified psychological care in improving patients’ emotional well-being, treatment adherence, and quality of life, while also reducing complications. By comparing this integrated nursing model with conventional nursing practices, the study seeks to provide evidence-based recommendations for optimizing nursing strategies in blood purification, ultimately enhancing patient outcomes and supporting recovery.

## METHODOLOGY

*Design.* This study was designed as a randomized controlled trial to evaluate the effects of nursing quality control combined with intensified psychological care on health outcomes of patients undergoing blood purification at the Affiliated Hospital of Jiangnan University from January 2021 to December 2023.

*Inclusion criteria*: 1) patients diagnosed as requiring blood purification treatment, including those with uremia, renal failure, poisoning, liver failure, or autoimmune diseases; 2) aged between 18 and 75 years; 3) the patient or their legal representative had signed the informed consent form, is willing to participate in the study, and agreed to receive nursing quality control and psychological care interventions; 4) had basic cognitive ability and could understand and comply with the research requirements; 5) had a relatively stable condition, was able to complete the prescribed course of blood purification, and could participate in long-term follow-up.

*Exclusion criteria*: 1) patients with severe complications (e.g., severe infection, acute heart failure, advanced malignant tumor) or unstable conditions that made them unable to tolerate blood purification; 2) patients with severe mental disorders or cognitive impairments that prevented cooperation with psychological assessment and nursing interventions; 3) pregnant or lactating women; 4) patients receiving other forms of psychological intervention or participating in other clinical studies at the time of this study; 5) patients with a history of poor treatment compliance or who are unable to complete follow-up; 6) patients receiving continuous blood purification treatment.

*Randomization.* Participants were randomly assigned to either the control or the intervention group using a computer-generated random number table. Written informed consent was obtained from all participants or their legal representatives before enrollment. The Ethics Committee of the Affiliated Hospital of Jiangnan University granted ethical approval (Approval Number: JNU2021-00173). This study strictly adhered to the ethical principles outlined in the Declaration of Helsinki.

To minimize potential bias, participants were informed that they would receive one of two standard care approaches, but the specific details of the intervention were not disclosed. Due to the nature of the intervention, the nursing staff delivering care could not be blinded. However, independent evaluators, blinded to group allocation, performed outcome assessments.

*Intervention*. The control group received routine blood purification nursing, including basic vital sign monitoring, execution of blood purification procedures, and standard psychological support.

The intervention group received nursing quality control and intensified psychological nursing in addition to routine nursing. Specific measures included:

- Establishment of a nursing quality control team: this team consisted of the department head nurse, blood purification nurses, and quality control specialists. Referring to the *Standard Operation of Blood Purification* and adapted to the hospital´s and department´s characteristics, the team developed and implemented protocols for pathological management, blood purification procedures, and hospital infection control.
- Standardization of nursing skills: nursing staff were required to strictly follow aseptic techniques and perform blood purification procedures in accordance with standardized procedures.
- Enhanced infection control: a 500 mg/L chlorine-containing disinfectant was used to thoroughly disinfect relevant medical equipment before and after each blood purification session.
- Quality supervision and feedback: the quality control team conducted regular monitoring of nursing procedures and analyzed feedback to continuously optimize nursing management.

Intensified psychological care included the following components:

- Comprehensive psychological assessment: the Hamilton Anxiety Scale (HAMA) and the Hamilton Depression Scale (HAMD) scores were administered upon hospital admission and discharged to assess the patient´s psychological state and quality of life.
- Personalized psychological support: care plans were according to patients´ age, gender, educational level, and medical condition. Young patients received virtual support (via WeChat or text messages), while elderly patients were visited bi-weekly by trained volunteers for companionship and emotional support.
- Diverse psychological intervention: 1) Relaxation training: conducted twice a week for 30 minutes, focusing on breathing exercises and guided imagery to reduce anxiety. 2) Music therapy: offered three times per week in 20-minute sessions, using calming music to promote emotional stability. 3) Cognitive- behavioral therapy (CBT): delivered weekly in 40-minute on one-on-one sessions by trained psychologists, targeting negative thought patterns and enhancing coping strategies. 4) Group activities: monthly lectures, mental health workshops, and peer support meetings were organized to encourage shared experiences and mutual support.
- Family psychological support: bi-weekly communication with family members to guide them in providing emotional support and encouragement to the patient. Family members also received psychological counseling to help them better cope with the patient’s condition.
- Psychological nursing records: a psychological care file was established for each patient, documenting assessment, intervention, and outcome evaluations. These records were regularly reviewed to summarize findings and continuously improve psychological care strategies.

Intervention duration: all psychological interventions were delivered consistently throughout the six-month study period. Adjustments to frequency or content were made based on patient and family feedback during follow-up evaluations.

### Measurements

Adverse emotion indicators: the HAMA and the HAMD scales were used to assess the severity of depression and anxiety, respectively.

- The HAMD consists of 21 items, with a total score range of 0-52. Scores are categorized as follows: 0-7: no depression, 8-17: mild depression, 18-22: moderate depression, and ≥23: severe depression^8^.
- The HAMA comprises 14 items, with a total score range of 0-56. Scores are categorized as follows: ≤17: mild anxiety, 18-24: mild to moderate anxiety, and 25-30: moderate to severe anxiety^8^.

Both scales demonstrated good original reliability, with Cronbach’s α value of 0.8 for the HAMD and 0.85 for the HAMA. These instruments have been linguistically and culturally validated for use in Chinese population^9^. In the current study sample, Cronbach’s α values were 0.82 and 0.83, respectively, indicating good internal consistency.

Quality of Life Assessment: the Short Form-36 Health Survey (SF-36)^10^ was used to evaluate patients’ quality of life. Four dimensions were assessed: physical functioning, role-physical, social functioning, and mental health. Each dimension is scored on a scale from 0 to 100 points, with higher scores indicating better health status. The original version of the SF-36 demonstrated reliability with Cronbach’s α ranging from 0.75 to 0.92. In this study, reliability remained strong, with Cronbach’s α ranging from 0.77 to 0.89^11^^-^^13^. The SF-36 has been validated in Chinese populations, and standardized scores were calculated based on normative Chinese data. Sample items included: *Have you felt sad or hopeless in the past week?* (HAMD) and *Have you felt full of energy in the past four weeks?* (SF-36).

*Treatment compliance*. Full compliance: patients strictly follow medical advice, actively cooperate with blood purification treatment, and adhere to a standardized treatment plan over time. Partial compliance: patients generally follow medical advice, though they may occasionally exhibit irregular treatment behavior. Non-compliance: patients frequently fail to follow medical instructions and do not adhere to standardized treatment. The total compliance rate was defined as the percentage of patients demonstrating either full or partial compliance.

*Complications*. The incidence of complications, such as infection, bleeding, and hypotension, was recorded for patients undergoing blood purification before and after nursing intervention.

### Data Analysis

Statistical analyses were performed using SPSS version 26.0 (IBM Corp, Armonk, NY, USA). Descriptive statistics were used to summarize baseline characteristics and study outcomes. Continuous variables are presented as mean ± standard deviation (SD) and compared using independent t-tests. Normality was assessed using the Shapiro-Wilk test, and homogeneity of variances was evaluated using Levene’s test. For non-normally distributed variables, the Mann-Whitney U test was applied. Categorical variables are presented as frequencies and percentages, with group differences analyzed using the Chi-square (χ²) test.

A power analysis was conducted to determine the minimum required sample size. Based on prior research on nursing interventions and assuming a medium effect size (Cohen’s d = 0.5), a significance level of 0.05, and a power of 80%, the minimum required sample size was estimated at 134 patients per group. To account for potential dropouts, 300 patients (150 per group) were recruited, ensuring adequate statistical power to detect significant inter-group differences.

## RESULTS

Three hundred patients were enrolled in the study, of whom 54.3% were female. Patients were randomly assigned to either the intervention group or the control group, with 150 participants in each. There were no statistically significant differences between the two groups in baseline characteristics, including gender, age, weight, heart rate, blood pressure, and etiology (Table 1).

**Table 1:** Baseline characteristic of patients receiving hemodialysisblood purification treatment randomized in control and intervention groups

Variable	Control	Intervention	p
	(n=150)	(n=150)	
Gender, *n (%)*			0.562
Male	84 (56.0)	79 (52.7)	
Female	66 (44.0)	71 (47.3)	
Age (years), *mean (SD)*	48.34 (7.62)	49.39 (8.25)	0.253
Range	42 - 56	43 - 56	
Weight (kg), *mean (SD)*	66.46 (8.28)	66.89. (7.56)	0.639
Heart rate (beats/min), *mean (SD)*	107.26 (31.29)	106.35 (33.87)	0.809
Blood pressure (mmHg), *mean (SD)*			
Systolic	123.75 (23.26)	124.66 (26.75)	0.753
Diastolic	68.92 (6.38)	68.75 (7.26)	0.829
Etiology, *n (%)*			0.474
Renal failure	53 (35.3)	59 (39.3)	
Acute pancreatitis	23 (15.3)	24 (16.0)	
Pyelonephritis	26 (17.3)	21 (14.0)	
Diabetes	48 (32.0)	46 (30.7)	

SD: standard deviation.

*Negative emotions.* Prior to the intervention, there were no significant inter-group differences in anxiety or depression scores (HAMA: p=0.177; HAMD: p=0.610). Following the treatment, both HAMA and HAMD scores were significantly lower in the intervention group compared to the control group (Table 2). Although both groups showed a significant reduction in anxiety and depression scores, the magnitude of improvement was notably greater in the intervention group: HAMA scores decreased by 42.66% vs 19.68% in the control group, and HAMD scores decrease by 37.55% versus 14.63%.

*Quality of life*. Post-treatment, the intervention group demonstrated significantly higher quality of life scores than the control group across all measured dimensions (p<0.001) (Table 2). Improvements from baseline were also greater in the intervention group: physical functioning (36.50 vs. 22.61), role-physical (36.99 vs. 23.26), social functioning (37.33 vs. 21.22), and mental health (34.48 vs. 18.95).

**Table 2:** Comparison of scores for negative emotions and quality of life between control and intervention groups

	Before		p	After		p
	Control Mean (SD)	Intervention Mean (SD)		Control Mean (SD)	Intervention Mean (SD)	
*Negative emotions*						
HAMA	36.54 (4.51)	37.25 (4.58)	0.177	29.35 (3.35)	21.36 (2.72)	<0.001
HAMD	32.39 (2.57)	32.54 (2.52)	0.610	27.65 (2.53)	20.32 (2.56)	<0.001
*Quality of life*						
Physical functioning	48.82 (3.18)	49.06 (4.32)	0.584	71.43 (7.45)	85.56 (7.67)	<0.001
Role physical	50.13 (4.43)	50.54 (4.76)	0.441	73.39 (7.75)	87.53 (7.84)	<0.001
Social functioning	50.53 (5.31)	50.94 (5.62)	0.517	71.75 (7.48)	88.27 (8.64)	<0.001
Mental health	53.18 (6.47)	52.87 (6.75)	0.685	72.13 (8.67)	87.35 (9.56)	<0.001

SD: standard deviation; HAMA: Hamilton anxiety scale; HAMD: Hamilton depression scale.

*Compliance*. After the intervention, the overall compliance rate in the intervention group was significantly higher than in the control group (98.67% vs. 70%). Specifically, the intervention group showed a higher rate of full compliance (64.67% vs 34%) and a lower rate of non-compliance (1.33% vs 30%). The rate of partial compliance was similar between the groups (Table 3).

*Complication rates.* The complication rate in the control group was 35.33%, significantly higher than the 10.00% observed in the intervention group (p<0.001). The most common complications in both groups were needle detachment (accounting for approximately one-third of the complications), followed by infection (about one-quarter), hypotension, and bleeding (Table 3).

**Table 3:** Comparison of compliance and complication rates

	Control	Intervention	p
	n (%)	n (%)	
*Compliance*	105 (70.00)	148 (98.67)	<0.001
Full	51 (34.00)	97 (64.67)	
Partial	54 (36.00)	51 (34.00)	
Non-compliant	45 (30.00)	2 (1.33)	
*Complications*	53 (35.33)	15 (10.00)	<0.001
Needle detachment	17 (11.33)	5 (3.33)	
Infection	14 (9.33)	4 (2.67)	
Hypotension	13 (8.67)	3 (2.00)	
Bleeding	9 (6.00)	3 (2.00)	

## DISCUSSION

This study demonstrates that integrating nursing quality control with intensified psychological care significantly enhances clinical outcomes in patients undergoing blood purification. Our findings reveal several key advantages of this dual-intervention strategy: (1) a marked reduction in anxiety and depression scores, with the intervention group achieving 42.7% and 37.6% reductions in HAMA and HAMD scores, respectively - substantially exceeding the 19.7% and 14.6% reductions observed in the control groups; (2) a comprehensive improvement in quality of life across all SF-36 domains, with physiological function increasing by 36.5 points in the intervention group versus 22.6 in the control group; (3) near-perfect treatment compliance rate (98.67% vs. 70.00%); and (4) a significantly lower complication rate (10.00% vs. 35.33%).

To further understand these clinical improvements, we explored potential biological mechanisms underlying the observed benefits. The reduction in anxiety and depression aligns with previous studies, such as those by Wang et al.^14^; however, our results indicate a greater magnitude of symptom relief. This enhanced effect may be attributed to the synergistic impact of structured quality control combined with continuous psychological support. Biologically, chronic psychological stress is known to aggravate systemic inflammation and dysregulate immune and neuroendocrine functions^15^^-^^18^. By alleviating emotional distress, our intervention likely reduced stress hormone levels (e.g., cortisol) and modulated inflammatory mediators, thereby supporting improved mental health outcomes^19^^-^^21^.

Beyond emotional improvements, the intervention led to significant gains in overall quality of life. The SF-36 increases across all domains (+34.5 to +37.3 points) surpassing those reported by Wang et al.^22^, possibly due to our dual emphasis on systemic quality assurance (e.g., environmental safety, procedural protocols, staff training) and patient-centered psychological care. From a biological standpoint, reductions in anxiety and depression may promote healthier behaviors and restore neuroendocrine balance, ultimately enhancing physical functioning, social engagement, and role fulfillment^23^^,^^24^.

Treatment compliance, another critical outcome, was also significantly improved by the intervention and likely contributed to the reduced rate in complications. The exceptionally high compliance rate (98.67%) reflects the effectiveness of comprehensive patient education, family involvement, and multidisciplinary collaboration. Biologically, improved adherence minimizes the risk of treatment disruption and reduces physiological stress during dialysis. For example, anxiety reduction may stabilize autonomic nervous system responses, thereby preventing complications such as dialysis-induced hypotension^25^^-^^27^. Simultaneously, strict adherence to aseptic techniques - reinforced by quality control frameworks - likely reduced infection-related complications, underscoring the broad benefits of the intervention.

Several limitations should be acknowledged. First, the single-center design may limit the generalizability of our findings; future multi-center studies involving populations that are more diverse are needed. Second, potential confounding factors, such as illness severity or baseline psychological status, were not fully controlled. Third, this study focused only on short-term outcomes; long-term follow-up is required to assess sustained effects on survival and overall well-being. Fourth, confidence intervals for the effect estimates were not reported, limiting the precision of our conclusions; future research should incorporate advanced statistical models to address this. Finally, survival analysis were not conducted, which would be valuable for assessing the intervention’s impact on mortality.

In conclusion, nursing quality control, combined with intensified psychological care, yields clinically meaningful benefits for patients undergoing blood purification. Compared to controls, patients in the intervention group experience significantly greater reductions in anxiety (42.7% vs. 19.7%) and depression (37.6% vs. 14.6%), greater improvement in quality of life (34.5-37.3 points across SF-36 domains), substantially higher compliance (98.67% vs. 70.00%), and fewer complications (10.00% vs. 35.33%). These findings suggest that a structured integration of psychosocial support and systemic quality oversight can substantially enhance patient outcomes. For effective implementation, hospitals should invest in nurse training, establish multidisciplinary care teams, and actively involve families in the treatment process. Future research should refine the optimal intensity and duration of such interventions, assess their long-term effect on survival, and explore their applicability in other high-stress clinical populations, such as patients undergoing chemotherapy or intensive care.

## Data Availability

The datasets used and analysed during the current study are available from the corresponding author on reasonable request.
